# A tale of (disordered) tail

**DOI:** 10.1038/s42003-023-04767-5

**Published:** 2023-04-14

**Authors:** Sumangal Roychowdhury, Krishnananda Chattopadhyay

**Affiliations:** 1 Structural Biology and Bioinformatics Division, CSIR-Indian Institute of Chemical Biology, Kolkata, 700 032 India; 2 Academy of Scientific and Innovative Research (AcSIR), Ghaziabad, 201002 India

**Keywords:** Biophysics

## Abstract

Although liquid-liquid phase separation (LLPS) has been extensively studied in various cellular and organismal contexts, the link between functional influence of a genetic mutation and LLPS with respect to human diseases is poorly understood. A recent article by Mensah et al. looks at a rare genetic disease to identify a frameshift mutation, which triggered aberrant phase separation and nucleolar dysregulation, linking genetic variants to a dysregulation of biomolecular condensates.

While cellular compartmentalization is traditionally achieved by cell membranes, recent studies in LLPS highlight the contribution of membraneless organelles. It has been shown that certain proteins, in the presence of nucleic acids or inside a crowded environment, can phase separate into dense (higher in concentration) and dilute phases (lower in concentration). Increasing evidence suggests that phase separation is not only the underlying mechanism towards the formation of stress granules, nucleolus, etc. but can be associated with transcription regulation, intracellular signalling, protein aggregation, and cellular fitness^1^. In a recent paper, Mensah et al. used a wide array of techniques to establish a link between aberrant phase separation of the protein HMGB1 and a rare genetic disease, brachyphalangy, polydactyly and tibial aplasia/hypoplasia syndrome (BPTAS)^2^. They identified de novo frameshift mutations in the *HMGB1* gene, which changed the intrinsically disordered acidic tail of HMGB1 into an arginine-rich basic tail resulting in an aberrant partitioning of the protein into the nucleolus, perturbing rRNA biogenesis (Fig. 1).

**Fig. 1:**
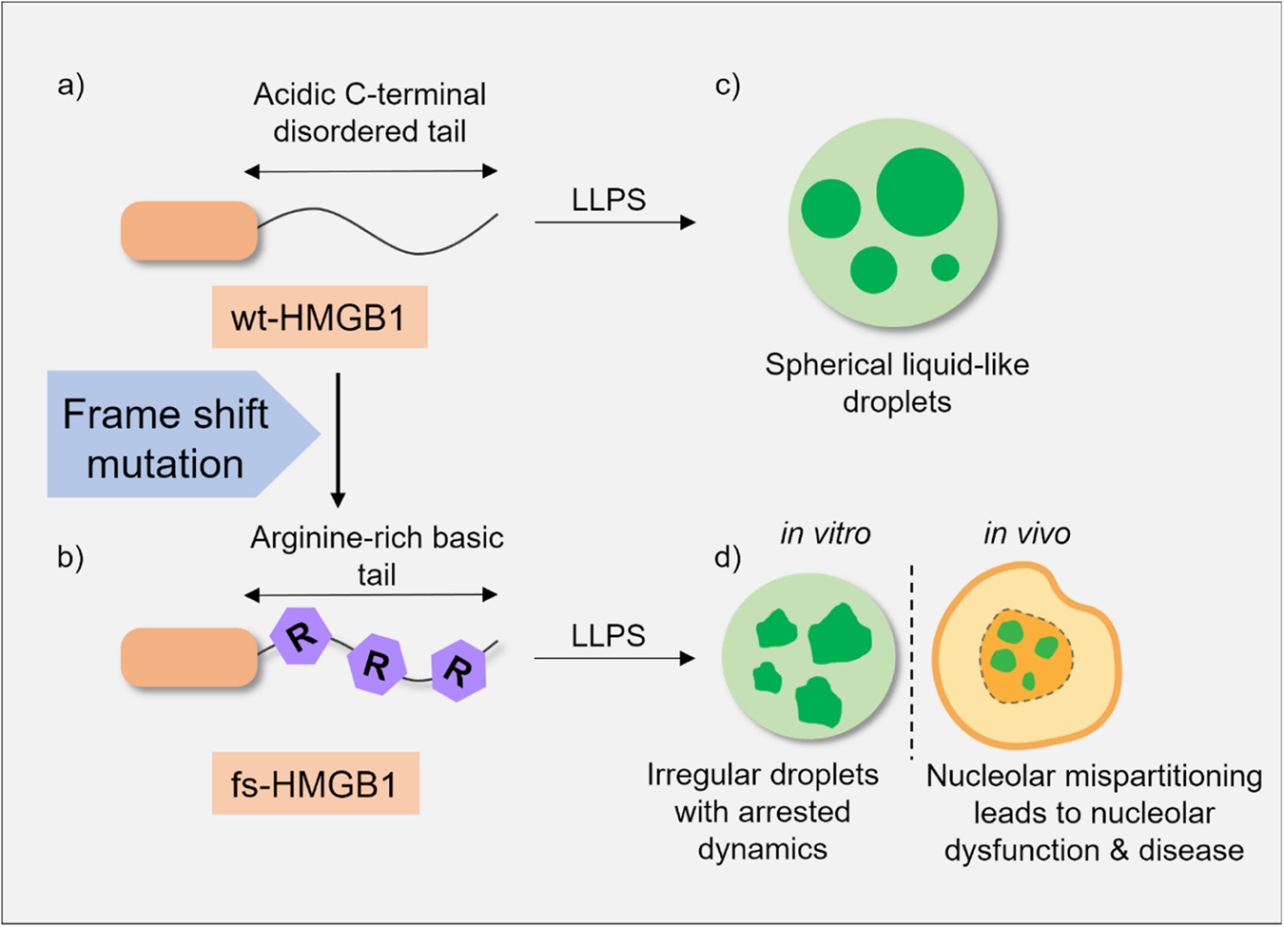
Schematic representation of HMGB1 phase separation and its link with BPTAS. **a** Cartoon representation of wt-HMGB1 with ~60 amino acids long acidic disordered tail. **b** Cartoon representation of fs-HMGB1 in which acidic tail is converted into an arginine-rich basic tail. **c** wt-HMGB1 phase separates into dynamic, spherical liquid droplets. **d** Aberrant phase separation of fs-HMGB1 produces dynamically arrested droplets both in vitro & in vivo. Credit: Sumangal Roychowdhury.

Genome sequencing using five BPTAS patients revealed involvement of potentially pathogenic frameshift mutants, namely E186Rfs^*^42 and K184Rfs^*^44. Structure predictions suggested the C-terminal tail of wt-HMGB1 (wild-type HMGB1) to be acidic and intrinsically disordered. In contrast, the same sequence region of fs-HMGB1 (the frameshift mutant) was predicted to be basic with helix-forming propensity. This change in conformation between the disordered tail and the helix was confirmed by far UV CD using synthetic peptides derived from the C-terminal region. Interestingly, phase separation assays using recombinantly expressed proteins revealed that the droplets of fs-HMGB1 appeared at low saturation concentration, showing low diffusion coefficient indicating arrested dynamics, when compared to wt-HMGB1 (Fig. 1). Complementary in vivo measurements using U2OS cells showed that fs-HMGB1 and fs-HMGB1 IDR (fs-HMGB1’s intrinsically disordered region only) were localized in the form of distinct nuclear inclusions containing cavities, resembling nucleoli-like multiphasic condensates. Co-partitioning experiments with other nucleolar proteins like NPM1 and FIB1 suggested that fs-HMGB1 inclusions were aberrantly phase separated nucleoli with arrested dynamics, which wrapped around a FIB1 core, replacing the NPM1-enhanced granular component of nucleoli. Arginine residues were found predominantly responsible for nucleolar mis-partitioning, whereas the hydrophobic patch in the C-terminal IDR contributed to the nucleolar arrest.

Importantly, mispartitioning of fs-HMGB1 lead to both nucleolar dysfunction by reducing ribosomal RNA (rRNA) biogenesis and cell death (Fig. 1). The authors established a global connection between the conformational switching at the C-terminal tail and nucleolar mispartitioning/dysfunction in other diseases using thirteen different proteins, which were identified by a detailed genomic analysis.

While the role of aberrant phase separation has been linked to neurodegeneration previously^1,3^, other disease systems are now being investigated. This study aims to provide a global model involving frameshift mutations, charge inversion at an intrinsically disordered region (IDR), and LLPS in rare human genetic diseases.
